# Crystal, Solution and *In silico* Structural Studies of Dihydrodipicolinate Synthase from the Common Grapevine

**DOI:** 10.1371/journal.pone.0038318

**Published:** 2012-06-25

**Authors:** Sarah C. Atkinson, Con Dogovski, Matthew T. Downton, F. Grant Pearce, Cyril F. Reboul, Ashley M. Buckle, Juliet A. Gerrard, Renwick C. J. Dobson, John Wagner, Matthew A. Perugini

**Affiliations:** 1 Department of Biochemistry, La Trobe Institute for Molecular Science, La Trobe University, Melbourne, Victoria, Australia; 2 Department of Biochemistry and Molecular Biology, Bio21 Molecular Science and Biotechnology Institute, The University of Melbourne, Victoria, Australia; 3 IBM Research Collaboratory for Life Sciences-Melbourne, Victorian Life Sciences Computation Initiative, Carlton, Australia; 4 Biomolecular Interaction Centre and School of Biological Sciences, University of Canterbury, Christchurch, New Zealand; 5 Department of Biochemistry and Molecular Biology, Monash University, Clayton, Victoria, Australia; 6 ARC Centre of Excellence in Structural and Functional Microbial Genomics, Monash University, Clayton, Victoria, Australia; University of Oulu, Finland

## Abstract

Dihydrodipicolinate synthase (DHDPS) catalyzes the rate limiting step in lysine biosynthesis in bacteria and plants. The structure of DHDPS has been determined from several bacterial species and shown in most cases to form a homotetramer or dimer of dimers. However, only one plant DHDPS structure has been determined to date from the wild tobacco species, *Nicotiana sylvestris* (Blickling *et al.* (1997) *J. Mol. Biol.* 274, 608–621). Whilst *N. sylvestris* DHDPS also forms a homotetramer, the plant enzyme adopts a ‘back-to-back’ dimer of dimers compared to the ‘head-to-head’ architecture observed for bacterial DHDPS tetramers. This raises the question of whether the alternative quaternary architecture observed for *N. sylvestris* DHDPS is common to all plant DHDPS enzymes. Here, we describe the structure of DHDPS from the grapevine plant, *Vitis vinifera*, and show using analytical ultracentrifugation, small-angle X-ray scattering and X-ray crystallography that *V. vinifera* DHDPS forms a ‘back-to-back’ homotetramer, consistent with *N. sylvestris* DHDPS. This study is the first to demonstrate using both crystal and solution state measurements that DHDPS from the grapevine plant adopts an alternative tetrameric architecture to the bacterial form, which is important for optimizing protein dynamics as suggested by molecular dynamics simulations reported in this study.

## Introduction

**Figure 1:**
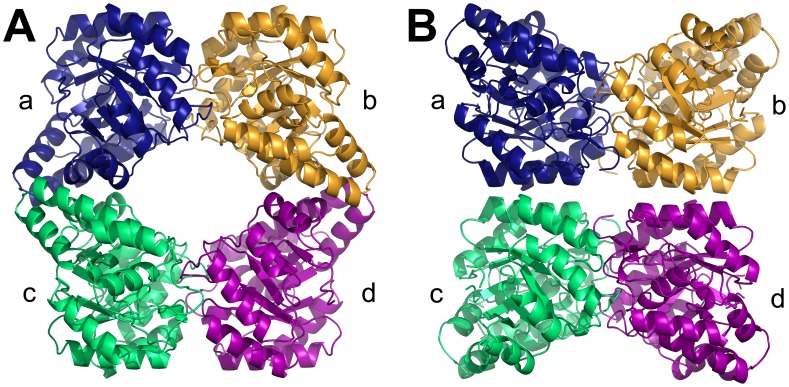
DHDPS from bacteria and plants. Dihydrodipicolinate synthase from (A) *B. anthracis* (PDB ID: 3HIJ [8]) and (B) *N. sylvestris* [25]. Structural coordinates of the *N. sylvestris* DHDPS were kindly provided by Prof Robert Huber (Max Planck Institute for Biochemistry).

Lysine is synthesized *de novo* in bacteria, plants and some fungi [1]–[3]. The lysine-biosynthesis pathway commences with the condensation of pyruvate and (*S*)-aspartate semialdehyde (ASA), to form (4*S*)-4-hydroxy-2,3,4,5-tetrahydro-(2*S*)-dipicolinic acid (HTPA) [1]–[4]. This reaction is catalyzed by dihydrodipicolinate synthase (DHDPS), which is the product of an essential gene in bacteria [1], [3], [5], [6]. The structure of DHDPS has been studied extensively from a number of bacteria, including *Bacillus anthracis* (*Ba*) [7], [8] (Fig. 1A), *Corynebacterium glutamicum* [9], *Escherichia coli* [10], [11], *Hahella chejuensis* [12], *Methanocaldococcus jannaschii* [13], *Mycobacterium tuberculosis* [14], *Neisseria meningitides* [15], *Pseudomonas aeruginosa* [16], *Staphylococcus aureus* [17], [18] and *Thermotoga maritima* [19]. Typically, bacterial DHDPS forms a tetramer of four identical (β/α)_8_-barrel monomers that can be described as a ‘head-to-head’ dimer-of-dimers (Fig. 1A). The tetramer contains four active sites, one per monomer, that are located at the ‘tight’ dimer interface (i.e. ab or cd, Fig. 1A). Each ‘tight’ dimer unit associates via noncovalent interactions at the ‘weak’ dimer interface (i.e. ac or bd, Fig. 1A) to form the homotetrameric structure. The ‘tight’ dimer interface also contains a cleft that binds the allosteric inhibitor, lysine, which mediates feedback inhibition in DHDPS enzymes from Gram-negative bacteria and plants [20]–[22]. However, DHDPS from Gram-positive species, including *Ba* [7], [8], [23] and *S. aureus* [17], [18] do not bind lysine and are thus insensitive to feedback inhibition. Given that the structural requirements for catalysis, and where appropriate allostery, are encoded by the ‘tight’ dimer unit, it is not obvious why the enzyme adopts a dimer-of-dimers. Interestingly, recent studies show that dimeric mutants of DHDPS from *E. coli* [5], [24] and *Ba* [8] possess significantly attenuated catalytic activity. Loss of function of the dimeric mutants is attributed to excessive dynamics or ‘breathing motion’ at the ‘tight’ dimer interface, which compromises the integrity of the active sites [5], [8]. Accordingly, the buttressing of two dimeric units together to form the homotetrameric structure is thought to stabilize the tight dimer interface, including the key active site residues [5], [8], [24].

By contrast, structural characterization of DHDPS from plants is limited to a single study of the enzyme from the wild tobacco plant, *Nicotiana sylvestris* [25]. This study shows that *N. sylvestris* DHDPS also forms a homotetramer, but in a ‘back-to-back’ arrangement (Fig. 1B) opposite in orientation to the typical bacterial tetrameric form (Fig. 1A). Consequently, the allosteric sites that bind lysine and mediate feedback inhibition [25] are located in the interior of the tetramer (Fig. 1B) rather than on the outside of the structure as observed for *E. coli* DHDPS [11]. However, the *N. sylvestris* enzyme [25] is the only plant DHDPS structure determined to date. The unique quaternary architecture observed in the crystal structure of *N. sylvestris* DHDPS has not yet been confirmed in other plant species or validated in aqueous solution; and surprisingly, the structural coordinates of the *N. sylvestris* enzyme are not available in the Protein Data Bank (PDB). Studies validating the quaternary structure of plant DHDPS will thus offer insight into the molecular evolution of this important oligomeric enzyme.

Therefore, the aim of this study was to determine the quaternary structure of DHDPS from the agriculturally-important species, *Vitis vinifera* (*Vv*) or the common grapevine. Here, we present a thorough characterization of the structure of *Vv*-DHDPS both in aqueous solution and the crystal state compared to *Ba*-DHDPS, an example of the typical bacterial tetramer (Fig. 1A). We show that *Vv*-DHDPS adopts a ‘back-to-back’ dimer-of-dimers consistent with the structure reported for *N. sylvestris* DHDPS (Fig. 1B), and subsequently demonstrate using molecular dynamics (MD) simulations that the ‘back-to-back’ architecture is important for stabilizing protein dynamics of the ‘tight’ dimer unit. This study suggests that DHDPS from plants adopt an alternative quaternary architecture to the typical bacterial form, thus offering insight into the molecular evolution of an important oligomeric enzyme.

## Results and Discussion

### Vv-DHDPS is Folded and Active

Recombinant *Vv*-DHDPS was expressed and purified to homogeneity as described previously [26]. Circular dichroism (CD) spectroscopy shows that the recombinant enzyme is folded (Fig. S1A) and is comprised of a similar proportion of α-helical and β-strand secondary structure as observed for DHDPS from other species [5], [8], [15], [17]. The CD spectrum of *Vv*-DHDPS was also obtained in the presence of the substrate, pyruvate. However, no significant change in secondary structure is observed in the presence of this ligand relative to the apo form (Fig. S1A). To confirm that the recombinant enzyme is active, the kinetic properties of *Vv*-DHDPS were determined using the quantitative coupled assay employing NADPH-dependent dihydrodipicolinate reductase (DHDPR) [27]. Initial rates (ΔAbs_340_ min^−1^) were measured at varying concentrations of both DHDPS substrates, pyruvate and ASA, using excess amounts of *E. coli* DHDPR and NADPH. The resulting Michaelis-Menten curves (Fig. S1B, symbols) were globally fitted to various bi-substrate kinetic models, namely the ternary complex, Ping-Pong and Ping-Pong with substrate inhibition models. The Ping-Pong model (without substrate inhibition) provided the global best fit (Fig. S1B, solid lines), which is consistent with the mechanism observed for other DHDPS enzymes [8], [21], [22], [28], [29]. The fit resulted in a R^2^ value of 0.98 and yielded *K*_M_ constants of 1.02 mM and 0.180 mM for pyruvate and ASA, respectively, and a *V*_max_ of 160 µmol min^−1^ mg^−1^ (*k*_cat_ = 45 s^−1^) (Fig. S1B). These kinetic constants are similar to those determined for other DHDPS enzymes [8], [29], [30].

### Vv-DHDPS is a Tetramer in Solution

**Figure 2:**
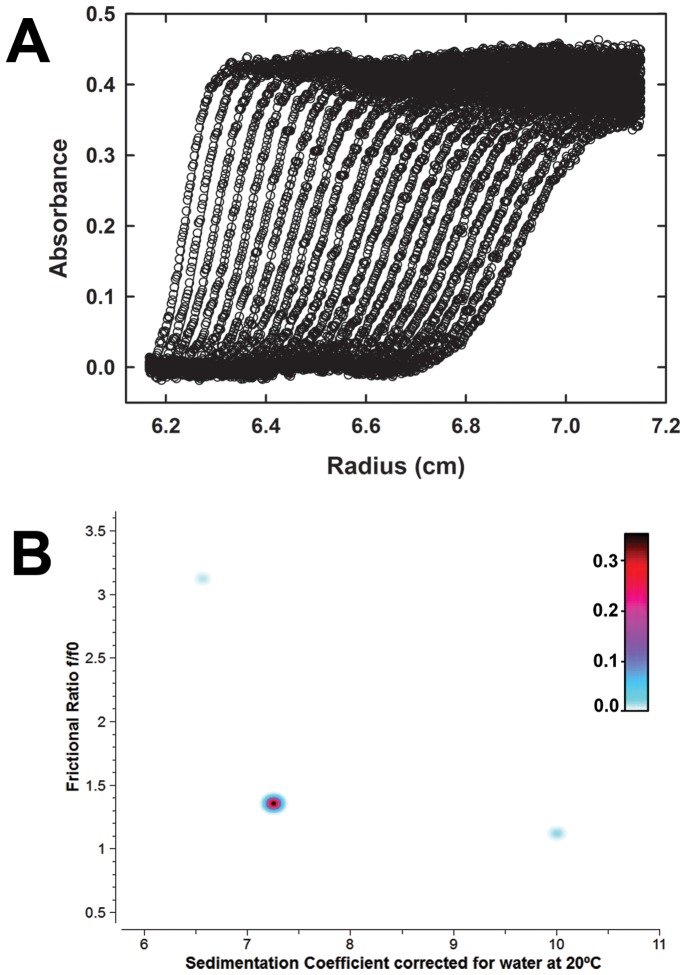
Sedimentation velocity analytical ultracentrifugation analysis of the quaternary structure of *Vv*-DHDPS in aqueous solution. (A) Absorbance at 280 nm measured as a function of radial position from the axis of rotation (cm) for *Vv*-DHDPS (13 µM) centrifuged at 40,000 rpm. The raw data are presented as open symbols plotted at time intervals of 10 min overlaid with the 2DSA fit shown in panel B. (b) Pseudo-3D plots of solute distributions for 2DSA Monte Carlo of *Vv*-DHDPS using a grid resolution of 10,000 solutes. The colour scale represents the signal of each species in optical density units.

To characterize the quaternary structure of *Vv*-DHDPS in aqueous solution, sedimentation velocity experiments were conducted in the analytical ultracentrifuge at an initial enzyme concentration of 13 µM in the presence and absence of pyruvate. The absorbance versus radial position profile for *Vv*-DHDPS in the absence of pyruvate is plotted in Fig. 2A. These data show a distinct sedimenting boundary consistent with the presence of a single species. This assertion is supported by 2-dimensional spectrum analysis (2DSA) [31], which decomposes the velocity experimental data into a sum of non-interacting finite element solutions and provides information on sedimentation and shape. The resulting analysis shows *Vv*-DHDPS (*M_r_* = 37,876.5) sediments predominantly as a single species with a standardized weight-average sedimentation coefficient (*s*_20,w_) of 7.3 S (Fig. 2B), molecular weight of 153 kDa (Fig. S2), and a frictional ratio (*f*/*f*_0_) of 1.35 (Table S1). These data demonstrate that *Vv*-DHDPS exists as a tetramer in aqueous solution. By contrast, *Ba*-DHDPS (*M_r_* = 31,233), which also forms a tetramer in solution and the crystal state [8], sediments with a *s*_20,w_ of 6.4 S (Table S1). Similar sedimentation velocity data were also obtained for *Ba*-DHDPS and *Vv*-DHDPS in the presence of saturating amounts of pyruvate (Fig. S3), which indicates that at an initial protein concentration of 13 µM the substrate does not alter the tetrameric quaternary structure of the enzymes. The hydrodynamic properties of *Vv*-DHDPS compared to *Ba*-DHDPS are summarized in Table S1.

### Crystal Structure of Vv-DHDPS Reveals Alternative Tetrameric Architecture

**Figure 3:**
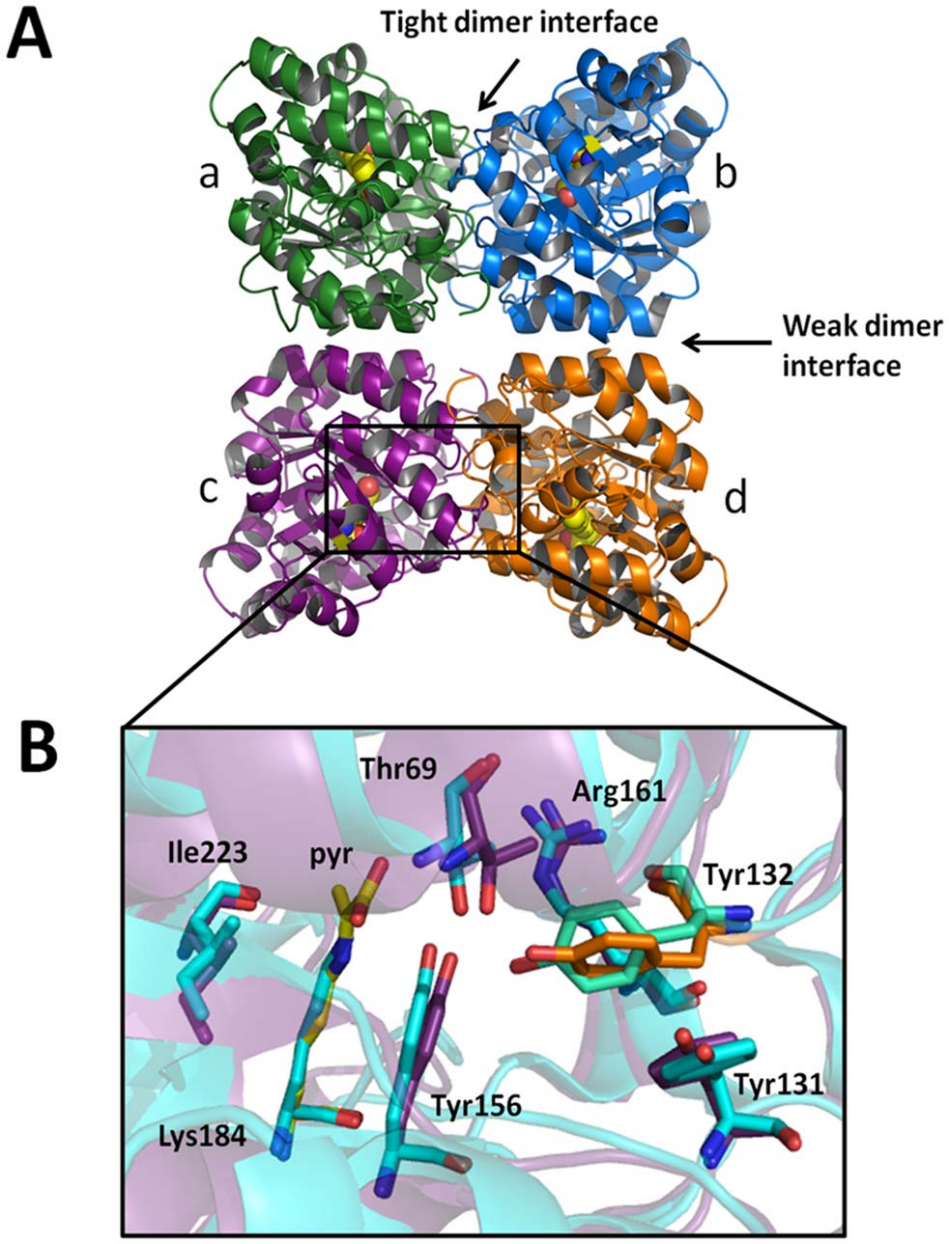
Crystal structure of *Vv*-DHDPS. (A) Crystal structure of *Vv*-DHDPS (PDB ID: 3TUU) showing the position of the active site lysine residue (yellow spheres) in each monomer and the self-association interfaces. Two monomers come together at the tight dimer interface to form the dimeric unit, which dock at the weak dimer interface to form a homotetramer. The asymmetric unit contained eight monomers assembled as two homotetramers. (B) Active site residues of *Vv*-DHDPS overlaid with *E. coli* DHDPS (cyan). Pyruvate is shown in yellow. Tyr132 (orange) from the adjacent monomer interdigitates across the tight interface and is overlaid with the equivalent residue in *E. coli* DHDPS (green).

To further investigate the quaternary structure and shape of the *Vv*-DHDPS tetramer, the crystal structure of the enzyme in complex with pyruvate was determined to a resolution of 2.2 Å (PDB ID: 3TUU) (Fig. 3A). The asymmetric unit contains two tetramers comprised of four identical subunits. Each tetramer can be described as a dimer-of-dimers, with the monomers within the two dimeric units ab and cd (Fig. 3A) tightly bound to each other, and weaker interactions between monomers ac and bd. The quaternary architecture of the *Vv*-DHDPS tetramer is identical to the *N. sylvestris* structure (Fig 1B) and quite distinctive from the typical bacterial tetramer (Fig 1A). Indeed, when crystal packing is investigated using symmetry operations, it can be seen that the orientation of the dimeric units are incompatible with formation of the bacterial head-to-head tetramer (Fig. S4).

Each monomer comprises an N-terminal (β/α)_8_-barrel domain and a C-terminal domain consisting of 3 α-helices and 2 short β-strands (Figs. S5A & S5B). The active site centers around Lys184, which forms a Schiff base with pyruvate and is located at the center of each monomeric unit (Fig. 3B). Three hydroxyl-containing amino acids, namely Tyr132, Thr69 and Tyr156, form the conserved catalytic triad with Tyr132 contributed from the adjacent monomer across the ‘tight’ dimer interface (i.e. interface between subunits ab or cd, Fig. 3A). The spatial orientation of the catalytic triad residues (Fig. 3B), as well as Arg161 and Ile223 that are also important for catalytic activity, is consistent with that observed in the active sites of other DHDPS structures [27], [30], [32]. Examination of potential interfaces within the enzyme using the *Protein Interfaces, Surfaces and Assemblies* (PISA) program [33] show that each of the two tight dimer interfaces (ab and cd) bury approximately 1790 Å^2^ per monomer, which corresponds to ∼14% of the total surface area of the monomer. By contrast, the interfaces between monomers a & c and b & d, bury a surface area of approximately 630 Å^2^ per monomer.

### SAXS Analyses Confirms Alternative Quaternary Structure of Vv-DHDPS

**Figure 4:**
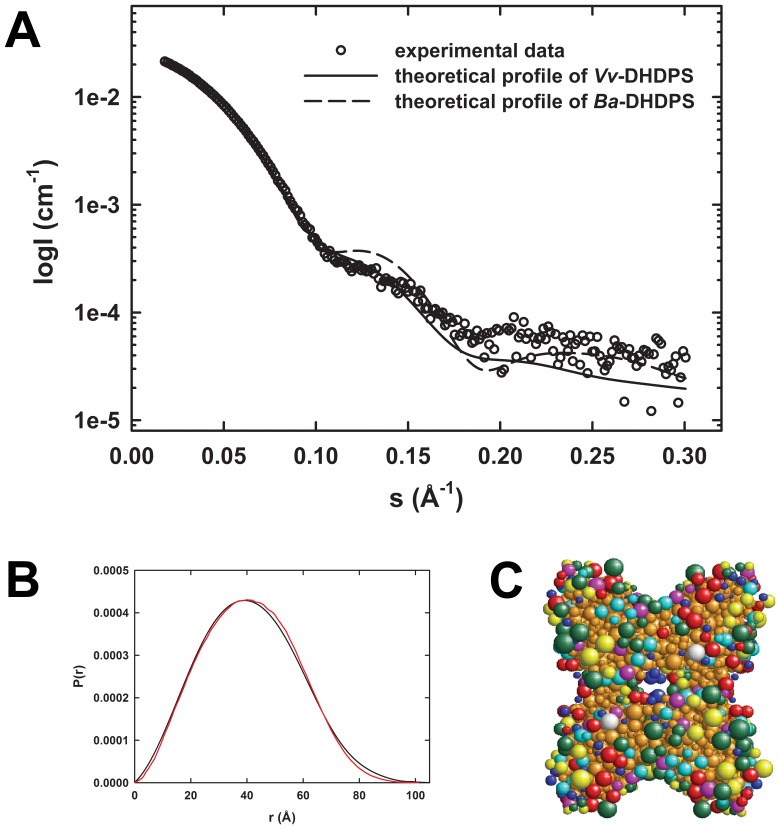
SAXS analyses of *Vv*-DHDPS. (A) Theoretical scattering profiles from *Vv*-DHDPS (solid line) and *Ba*-DHDPS (dashed line) and the raw SAXS data (○).Theoretical scattering profiles were generated from crystallographic coordinates using CRYSOL. (B) *P*(r) plots of *Vv*-DHDPS from experimental data (black) and SOMO bead model (red) using ULTRASCAN. (C) SOMO bead model of *Vv*-DHDPS. The various colored beads represent acidic (green), hydrophobic (cyan), polar (red), basic (yellow) and non-polar (magenta) side-chains. Blue beads represent the protein main-chain and brown indicates buried beads.

SAXS was employed to validate the analytical ultracentrifugation studies in solution (Fig. 2) and the crystal structure of *Vv*-DHDPS (Fig. 3). Scattering data for *Vv*-DHDPS were collected in the presence of pyruvate (Fig 4A), compared to *Ba*-DHDPS (Fig. S6A).The radius of gyration (*R*_g_) for *Vv*-DHDPS was determined by Guinier analysis to be 35.2 Å. The pair distance distribution function [P(r)] was calculated using the indirect Fourier transform method (Fig. 4B). The *R*_g_ from the P(r) analysis plot was calculated to be 34.3 Å, and the maximum dimension of the scattering particle (*D*_max_) to be 100 Å, which is in close agreement with the crystal structure (Fig. 3A, *D*_max_ is 100 Å). By comparison, *Ba*-DHDPS has a *R*_g_ of 30.9 Å determined by Guinier analysis and a *R*_g_ of 31.7 Å with a *D*_max_ of 90 Å determined from P(r) analysis (Fig. S6C). Again, this is in close agreement with the crystal structure (PDB ID: 3HIJ, *D*_max_ is 85 Å). The scattering profile data for *Ba*-DHDPS is consistent with the tetrameric form of the protein observed in the crystal structure and in solution (Fig. S6A) [8].

Direct comparisons of both the crystal structures and SAXS profiles of *Vv*-DHDPS and *Ba*-DHDPS (PDB ID: 3HIJ [8]) were performed using CRYSOL [34]. The scattering profile of *Vv-*DHDPS fits that calculated for the crystal structure of the enzyme (Fig. 4A) [reduced chi-squared (χ^2^_v_) = 1.5]. This represents a statistically better fit to the data than the fit to the *Ba*-DHDPS CRYSOL profile [(χ^2^_v_ = 7) (*P_F_*(*F*;*v*_1_,*v*_2_) <8.7×10^−34^ (*F* >4.5, *v*_1_ = 278, *v*_2_ = 278)] [35]. The crystal structure and theoretical scattering profile of *Vv*-DHDPS do not exactly overlay, which is indicative that there are some differences between the protein in solution compared to the crystal state. Indeed, crystal packing (Fig. S4) and the high concentrations of polyethylene glycol and salt employed for crystallization [26] may account for this difference. In addition, the lack of electron density observed for the N-terminal region (including the His-tag) indicates that this region is highly flexible. Thus, CORAL [36] rigid body modeling was performed using the *Vv*-DHDPS PDB coordinates (3TUU) with the 30 missing N-terminal residues added. This yielded a significantly better fit (Fig. S7). Conversely, the *Ba*-DHDPS scattering data fit more closely to the theoretical profile calculated from the crystal structure of *Ba*-DHDPS (3HIJ) when analyzed by CRYSOL (χ^2^_v_ = 1.2), compared to that for *Vv*-DHDPS (χ^2^_v_ = 6.3) (Fig. S6A). In addition, the *Vv*-DHDPS and *Ba*-DHDPS crystal structures were also used to construct bead models (Fig. 4C and Fig. S6B) using the program SOMO in the ULTRASCAN software package [37]. The resulting theoretical P(r) distributions fit well to the experimental P(r) distributions (Fig 4B and Fig. S6C). The SOMO bead models were also used to predict the hydrodynamic properties from the crystal structures of *Ba*-DHDPS (Fig. 1A) and *Vv*-DHDPS (Fig. 3A), which agree well with those determined by analytical ultracentrifugation (Table S1). The higher than predicted frictional ratio determined by analytical ultracentrifugation for *Vv*-DHDPS (Table S1) is likely to be due to the N-terminal region of the enzyme that is disordered and thus absent in the crystal structural model shown in Fig. 3A. Nevertheless, analytical ultracentrifugation, X-ray crystallography and SAXS analyses together demonstrate that *Vv*-DHDPS forms a ‘back-to-back’ tetrameric architecture (Figs. 2, 3, and 4), compared to the ‘head-to-head’ conformation observed for the typical bacterial tetramer (Fig. 1A & Fig. S6).

### Molecular Dynamics Simulations of Vv-DHDPS

**Figure 5:**
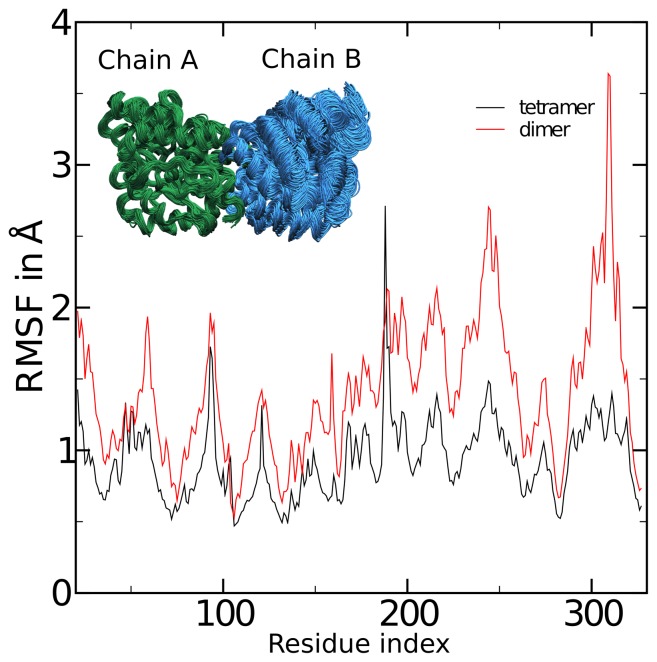
Comparison of the molecular dynamics of the native tetramer and a putative dimeric form of *Vv*-DHDPS. Simulations were analyzed by aligning chain A from all frames of the trajectories, and computing the root mean squared fluctuations (RMSF) of chain B, the monomer on the opposite side of the ‘tight-dimer’ interface. Shown are the RMSF values by residue number for the dimer (red) and tetramer (black). The inset shows 75 frames of the aligned dimer at 1 ns intervals.

To gain insight into the importance of tetramerization to the *Vv*-DHDPS structure, molecular dynamics (MD) simulations were performed. MD simulations were conducted on the *Vv*-DHDPS tetramer (i.e. chains a, b, c & d, Fig. 3A) compared to the ‘tight’ dimer unit (i.e. chains ab, Fig. 3A). The MD simulations show that the majority of residues in the *Vv*-DHDPS ‘tight’ dimer have significantly greater root mean square fluctuations (RMSFs) (Fig. 5, red line) compared to the wild-type tetramer (Fig. 5, black line). The larger RMSFs observed for the dimer include the key catalytic residues Thr69, Tyr156 and Lys184, as well as Tyr132, which interdigitates across the ‘tight’ dimer interface to form part of the active site of the adjacent monomer (Fig. 5). These data suggest that the dimer possesses greater conformational flexibility than the wild-type tetramer, and thus formation of the ‘back-to-back’ dimer-of-dimers functions to attenuate protein dynamics. Indeed, similar MD simulation results have recently been reported for bacterial DHDPS [38], which in turn, support biophysical analyses of mutant dimers and wild-type tetramers from the bacterial species *Ba* [8] and *E. coli* [5], [24]. Taken together, the results presented here and those reported in previous studies [5], [8], [24], [38], suggest that plant and bacterial DHDPS enzymes evolved to form homotetramers, albeit with different quaternary architectures, as a means to attenuate ‘breathing motion’ of the ‘tight’ dimer unit. This study therefore offers further insight into the molecular evolution at the quaternary structure level of an important bacterial and plant enzyme.

### Conclusions

In this study, we show for the first time that DHDPS from the plant *Vitis vinifera* forms a homotetramer or dimer-of-dimers both in solution and the crystal state (Figs. 2, 3, and 4). Consistent with previous studies of DHDPS from *Nicotiana sylvestris* [25], we demonstrate that *Vv-*DHDPS adopts a ‘back-to-back’ dimer-of-dimers compared to the ‘head-to-head’ architecture observed for DHDPS from most bacterial species. We subsequently show using MD simulations that tetramerization of *Vv-*DHDPS is important for attenuating protein dynamics of the ‘tight’ dimer unit, which offers insight into the molecular evolution of an important bacterial and plant enzyme.

**Table 1:** Data collection, processing and refinement statistics for *Vv*-DHDPS (PDB ID: 3TUU).

Wavelength (Å)	0.9536
No. of images	720
Step range (°)	0.5
Space group	*P*1
Unit cell parameters (Å)	a = 70.6, b = 78.9, c = 135.4
Bond angles (°)	α = 93.19, β = 95.02, γ = 100.61
Resolution (Å)	59-2.2 (2.26-2.20)
Observed reflections	470,484 (69,507)
Unique reflections	123,307 (18,027)
Completeness (%)	97.4 (97.3)
*R*_merge_ †	0.108 (0.454)
*R*_r.i.m_ ‡	0.126 (0.527)
*R*_p.i.m_ §	0.64 (0.267)
Mean I/σ (I)	10.4 (3.1)
Redundancy	3.8 (3.9)
Wilson B value	22.33
Molecules per ASU	8
*V*_M_ (Matthews coefficient)	2.55
Solvent content (%)	52
**R_cryst_**	0.196
**R_free_**	0.226
**Number of atoms**	19996
** Protein**	18880
** Water**	1155
** Ions**	23
**Rmsd**	
** Bonds**	0.010
** Angles**	1.337
**Average B factors**	
** Protein**	23.224
** Water**	28.163
**Ramachandran plot, # residues (%)**	
** Favored region**	97.99
** Allowed region**	1.58
** Disallowed region**	0.42

Values in brackets are for the highest resolution bin.

† R_merge_ = 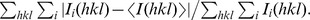

‡ R_r.i.m_ = 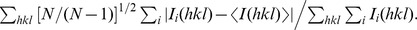

§ R_pim_ = 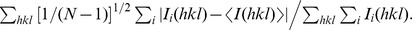

where 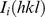 is the *i*th intensity measurement of reflection *hkl* and 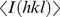 its average and N is the redundancy of a given reflection.

## Materials and Methods

### Cloning, Expression and Purification of Vv-DHDPS

The *dapA* gene encoding *Vv*-DHDPS was purchased from Geneart and cloned into the pET28a expression vector as described elsewhere [26]. Recombinant protein was produced in the host strain *E. coli* BL21-DE3 via induction by IPTG at 16°C. Cells were harvested following overnight IPTG treatment and then resuspended in 20 mM Tris-HCl, pH 8.0, 500 mM NaCl, 20 mM imidazole, before lysis by sonication. *Vv*-DHDPS was subsequently isolated by metal-affinity liquid chromatography as described previously [26].

### Circular Dichroism Spectroscopy

Circular dichroism (CD) spectra of *Vv*-DHDPS (4 µM) were recorded using an Aviv Model 410-SF CD spectrometer. Wavelength scans were performed between 198 and 250 nm in 20 mM Tris, 150 mM NaCl, pH 8.0 in 1.0 mm quartz cuvette as reported previously [8], [17], [39]. Data were analysed using the CDSSTR algorithm from the CDPro software package [40] incorporating the SP22X database.

### DHDPS-DHDPR Coupled Enzyme Kinetic Assay

Kinetic analyses of *Vv*-DHDPS were performed using the DHDPS-DHDPR coupled assay as previously described [27], using *E. coli* DHDPR. Assays were routinely conducted in triplicate at a constant temperature of 30°C with reaction mixtures allowed to equilibrate in a temperature-controlled Cary 4000 UV-visible spectrophotometer for 10 min before initiating the reaction with 60 nM DHDPS. Prior to the experiment, pyruvate and ASA concentrations were routinely quantified by the addition of limiting amounts of substrate by measuring the oxidation of NADPH spectrophotometrically at 340 nm. Initial rate data were analyzed using the ENZFITTER program available from Biosoft. Data were fitted to the various models, including the bi-bi ping-pong substrate model that yielded the best fit as assessed by Sigma values and the lowest standard error associated with the kinetic constants.

### Analytical Ultracentrifugation

Sedimentation velocity experiments were performed in a Beckman Coulter model XL-I analytical ultracentrifuge. Double sector quartz cells were loaded with 400 µL of buffer and 380 uL of *Vv*-DHDPS or *Ba*-DHDPS at an initial concentration of 13 µM. The cells were loaded into an An50-Ti rotor and left to equilibrate at 30°C. The rotor was accelerated to 40,000 rpm and absorbance readings were collected continuously at 280 nm and 30°C using a step size of 0.003 cm without averaging. Initial scans were carried out at 3,000 rpm to determine the optimal wavelength and radial positions for the high speed experiment. Samples of *Vv*-DHDPS monitored in the presence of pyruvate contained ligand in both the reference and sample channels. Solvent density, solvent viscosity, and estimates of the partial specific volume of *Vv*-DHDPS (0.7386 ml/g) and *Ba*-DHDPS (0.7463 ml/g) at 30°C were calculated using SEDNTERP [41]. Data were analyzed via 2-Dimensional Spectrum (2DSA) Monte Carlo analysis [31] incorporated in the ULTRASCAN software package [42], [43], which can be downloaded from www.ultrascan.uthscsa.edu.

### Crystallization of Vv-DHDPS and X-ray Diffraction Data

*Vv*-DHDPS was crystallized as described previously using sitting- and hanging-drop vapor diffusion [26]. For X-ray data collection, crystals were transferred to reservoir solution containing 20% (v/v) glycerol and directly flash frozen in liquid nitrogen. Intensity data were collected at the Australian Synchrotron using the MX2 beamline as described in [26]. Diffraction data sets were processed and scaled using the package MOSFLM [44] and SCALA [45], [46]. Molecular replacement was performed using PHASER [47] with *E. coli* DHDPS (PDB ID: 1YXC [11]) as the search model. CHAINSAW [48] from the CCP4 suite [46] was used to prepare the model of *E. coli* DHDPS, omitting waters and reducing it to its monomeric form. Structural refinement of the resulting 8 monomers was performed using REFMAC5 [46],[49] with iterative model building using COOT [50]. In the first steps of refinement, non-crystallographic restraints were applied, followed by simulated annealing using PHENIX [51]. The structure was validated using the MolProbity Server [52]. Refinement statistics are given in Table 1. Ramachandran statistics showed 98% of the residues in the most favored region, 1.6% in the additionally allowed regions and 0.4% (a single residue) in the disallowed region, namely Tyr 132, which is consistent with the equivalent Tyr residues observed in DHDPS structures from other species [7], [11].

### Small Angle X-ray Scattering

Small-angle X-ray scattering (SAXS) data were collected at the Australian Synchrotron, Clayton on the SAXS/WAXS beamline. The X-ray beam size at the sample was 250 µm horizontal, 80 µm vertical and data were collected using a Pilatus 1M detector positioned 900 mm from the sample, giving a q range of 0.01–0.6 Å^−1^ (wavelength, 1.0332 Å). The protein sample analyzed was subjected to in-line size exclusion chromatography on a Superdex 200 5/150 GL gel-filtration column (GE Healthcare) with a bed volume of 3 ml equilibrated with buffer at a flow rate of 0.2 ml.min^−1^. 50 µl *Vv*-DHDPS at 234 µM was injected and the fractionated sample flowed through a 1.5 mm quartz capillary where it was exposed to the X-ray beam. 600 detector images of sequential 5 s exposures were collected at 298 K, corresponding to a total elution volume of 4.2 ml. Radial averaging, background subtraction and image-series analysis were performed using SAXS15ID software (Australian Synchrotron). Eight sequential detector images were averaged to generate each SAXS data set for subsequent analysis using the ATSAS (v.2.3) software [53]. The region of the gel filtration chromatogram used for analysis is shown in Fig. S8. Guinier fits were made using PRIMUS [54] and P(r) distribution analyses performed using GNOM [55]. Theoretical scattering curves were generated from atomic coordinates and compared with experimental scattering curves using CRYSOL [34] and CORAL [36]. Statistical analysis was performed as described previously [35]. Briefly, for each fit 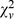 was calculated from 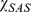, the parameter reported by CRYSOL.
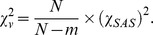
where *N* is the number of data points and *m* the number of fitting parameters. To analyze a change in 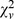 between the *Ba*-DHDPS and *Vv*-DHDPS fits, the statistic 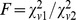 was calculated. The *F* distribution was then integrated to yield the probability the two 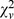 values are equal, 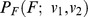, where 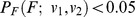 indicates significance. Bead models and subsequent P(r) plots were generated using the Solution Modeler (SOMO) software [37] in the ULTRASCAN suite [42], [43].

### Molecular Dynamics Simulations

Molecular dynamics simulations were performed with the native *Vv*-DHDPS tetramer from the crystal structure (PDB ID: 3TUU) and a dimer formed from two monomers joined at the ‘tight’ dimer interface. In both cases, the substrate, pyruvate, was removed and the structures were solvated using the TIP3P water model. The CHARMM force field [56] and the molecular dynamics package NAMD [57] were used. After allowing 25 ns for the structure to equilibrate at constant temperature of 293K and atmospheric pressure, trajectories of 75 ns were generated at 0.01 ns intervals.

## Supporting Information

Figure S1 **Secondary structure and enzyme kinetic analyses of** ***Vv*** **-DHDPS.** (A) CD spectra of *Vv*-DHDPS at 0.2 mg/ml recorded in 0.5 nm increments with a 2 s averaging time from 198 to 250 nm. Samples were prepared in standard buffer and analyzed in a 1 mm pathlength quartz cuvette. Raw data without pyruvate (circles) and with 5 mM pyruvate (triangles) were fitted by nonlinear least squares regression (solid lines) using the CDPro software package and employing the CDSSTR algorithm with the SP22X reference set [40]. The nonlinear best fit resulted in a RMSD of 0.145 and structural composition of 30% α-helix, 20% β-strand, 28% turn and 22% unordered structure for the absence of pyruvate and a RMSD of 0.121 and structural composition of 31% α-helix, 18% β-strand, 24% turn and 27% unordered structure in the presence of pyruvate. (B) Michaelis-Menten analyses of *Vv*-DHDPS. The initial velocity at 0.1–3.0 mM pyruvate plotted as a function of ASA concentration (dots). A global best-fit to a bi-bi Ping Pong model without substrate inhibition using the ENZFITTER software package (BioSoft) with an R^2^ of 0.98 and p>F of 9.37×10^−39^.
(TIF)

Figure S2 **Sedimentation velocity molecular weight analysis of** ***Vv*** **-DHDPS.** Pseudo 2DSA plot of *f*/*f*_0_ versus molecular weight of *Vv*-DHDPS using the data shown in Figure 2A. A grid resolution of 10,000 solutes was employed [31]. The colour scale represents the signal of each species in optical density units.
(TIF)

Figure S3 **Sedimentation velocity analyses of** ***Ba*** **-DHDPS and** ***Vv*** **-DHDPS in the presence of pyruvate.** Shown are the pseudo-3D plots for solute distributions for 2DSA Monte Carlo analyses of *Ba*-DHDPS (panel A) and *Vv*-DHDPS (panel B) at an initial protein concentration of 13 µM in the presence of 5 mM pyruvate. A grid resolution of 10,000 solutes was employed in the analyses [31]. The colour scale represents the signal of each species in optical density units.
(TIF)

Figure S4 **Crystal lattice of** ***Vv*** **-DHDPS (PDB ID: 3TUU).** *Vv*-DHDPS crystal packing generated using symmetry operations. The orientation of the dimeric units is incompatible with formation of the head-to-head tetramer commonly observed in bacteria DHDPS (Fig. 1A).
(TIF)

Figure S5 **Tertiary structure of** ***Vv*** **-DHDPS (PDB ID: 3TUU).** (A) View looking down the (β/α)_8_-barrel and C-terminal domain. The active site is defined by the position of Lys184 (stick view). (B) Side view of the (β/α)_8_-barrel and C-terminal domain.
(TIF)

Figure S6 **SAXS analyses of** ***Ba*** **-DHDPS.** (A) Fits of theoretical scattering profiles from *Vv*-DHDPS (dashed line) and *Ba*-DHDPS (solid line) to the SAXS data (▴). Theoretical scattering profiles were generated from crystallographic coordinates and fitted to the *Ba*-DHDPS SAXS data using CRYSOL [36]. (B) SOMO bead model of *Ba*-DHDPS. The various colored beads represent acidic (green), hydrophobic (cyan), polar (red), basic (yellow) and non-polar (magenta) side-chains. Blue beads represent the protein main-chain and brown indicates buried beads. (C) *P(r)* plots of *Ba*-DHDPS from experimental data (black) and SOMO bead model shown in panel B (blue) (37) using ULTRASCAN [42], [43].
(TIF)

Figure S7 **Comparison of experimental and theoretical SAXS data of** ***Vv*** **-DHDPS.** Theoretical scattering profile from *Vv*-DHDPS (solid line) with 30 N-terminal residues modeled using CORAL (36) to the SAXS data (•). This fit (reduced chi-squared (χ^2^_v_) = 1.0). This represents a statistically better fit to the data than the fit to the CRYSOL profile [(χ^2^_v_  = 1.5) (*P_F_*(*F*;*v*_1_,*v*_2_) <5.1 × 10^−11^ (*F* >2.2)].
(TIF)

Figure S8 **SAXS gel filtration chromatogram.** Fraction employed in SAXS analysis is highlighted in pink.
(TIF)

Table S1 **Hydrodynamic properties of** ***Vv*** **-DHDPS and** ***Ba*** **-DHDPS.**
(DOCX)
