# Supplementary figures and images for: Crystal, Solution and In silico Structural Studies of Dihydrodipicolinate Synthase from the Common Grapevine
Source: PLoS One. 2012 Jun 25;7(6):e38318. doi: 10.1371/journal.pone.0038318 (PMC3382604; doi:10.1371/journal.pone.0038318)

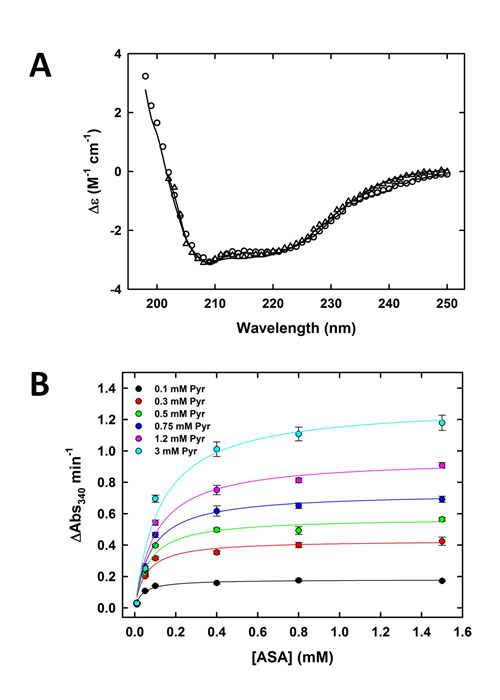

Supplement: Figure S1 — Secondary structure and enzyme kinetic analyses of Vv -DHDPS. (A) CD spectra of Vv-DHDPS at 0.2 mg/ml recorded in 0.5 nm increments with a 2 s averaging time from 198 to 250 nm. Samples were prepared in standard buffer and analyzed in a 1 mm pathlength quartz cuvette. Raw data without pyruvate (circles) and with 5 mM pyruvate (triangles) were fitted by nonlinear least squares regression (solid lines) using the CDPro software package and employing the CDSSTR algorithm with the SP22X reference set [40]. The nonlinear best fit resulted in a RMSD of 0.145 and structural composition of 30% α-helix, 20% β-strand, 28% turn and 22% unordered structure for the absence of pyruvate and a RMSD of 0.121 and structural composition of 31% α-helix, 18% β-strand, 24% turn and 27% unordered structure in the presence of pyruvate. (B) Michaelis-Menten analyses of Vv-DHDPS. The initial velocity at 0.1–3.0 mM pyruvate plotted as a function of ASA concentration (dots). A global best-fit to a bi-bi Ping Pong model without substrate inhibition using the ENZFITTER software package (BioSoft) with an R2 of 0.98 and p>F of 9.37×10^−39^. (TIF) [file pone.0038318.s001.tif]

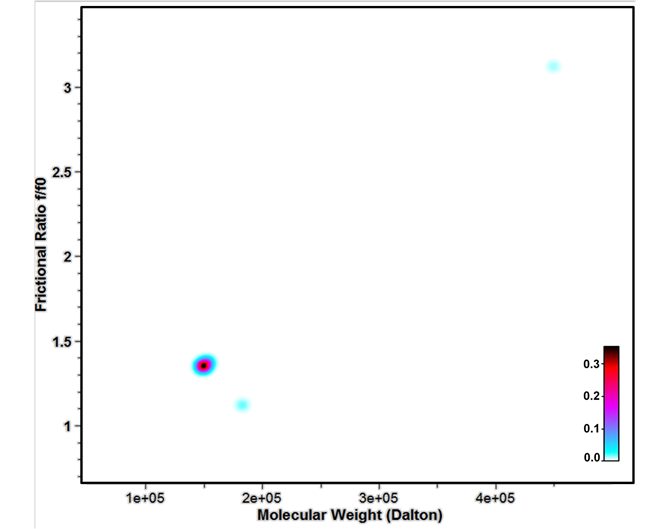

Supplement: Figure S2 — Sedimentation velocity molecular weight analysis of Vv -DHDPS. Pseudo 2DSA plot of f/f 0 versus molecular weight of Vv-DHDPS using the data shown in Figure 2A. A grid resolution of 10,000 solutes was employed [31]. The colour scale represents the signal of each species in optical density units. (TIF) [file pone.0038318.s002.tif]

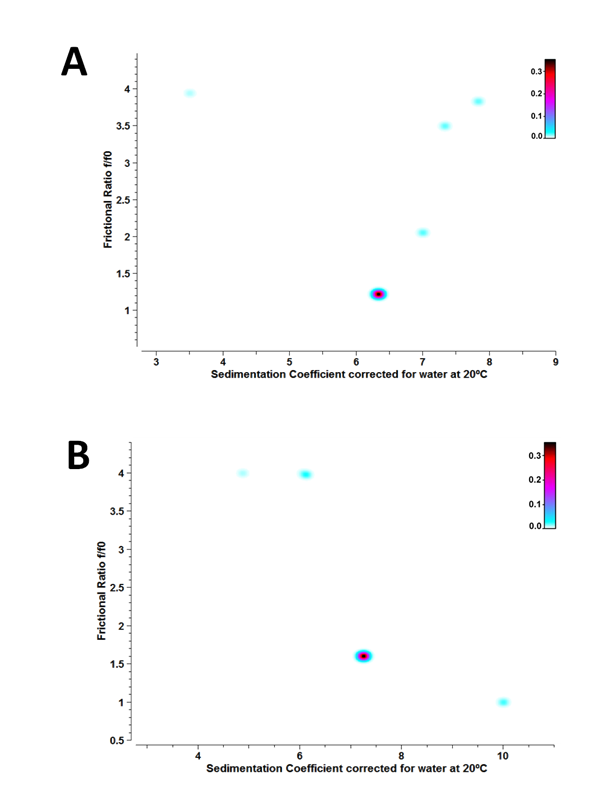

Supplement: Figure S3 — Sedimentation velocity analyses of Ba -DHDPS and Vv -DHDPS in the presence of pyruvate. Shown are the pseudo-3D plots for solute distributions for 2DSA Monte Carlo analyses of Ba-DHDPS (panel A) and Vv-DHDPS (panel B) at an initial protein concentration of 13 µM in the presence of 5 mM pyruvate. A grid resolution of 10,000 solutes was employed in the analyses [31]. The colour scale represents the signal of each species in optical density units. (TIF) [file pone.0038318.s003.tif]

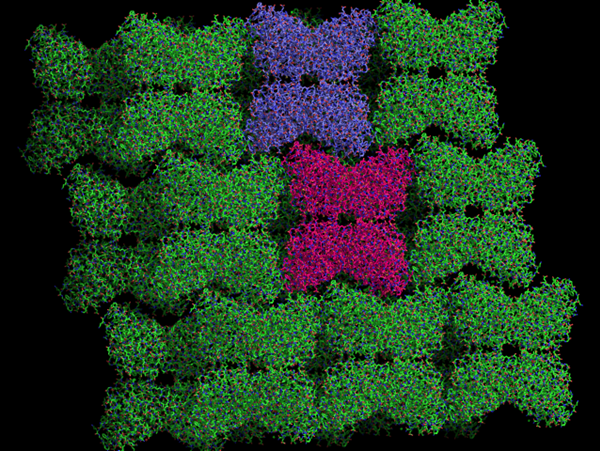

Supplement: Figure S4 — Crystal lattice of Vv -DHDPS (PDB ID: 3TUU). Vv-DHDPS crystal packing generated using symmetry operations. The orientation of the dimeric units is incompatible with formation of the head-to-head tetramer commonly observed in bacteria DHDPS (Fig. 1A). (TIF) [file pone.0038318.s004.tif]

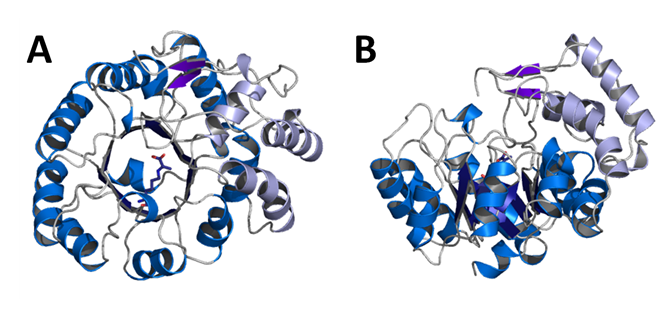

Supplement: Figure S5 — Tertiary structure of Vv -DHDPS (PDB ID: 3TUU). (A) View looking down the (β/α)8-barrel and C-terminal domain. The active site is defined by the position of Lys184 (stick view). (B) Side view of the (β/α)8-barrel and C-terminal domain. (TIF) [file pone.0038318.s005.tif]

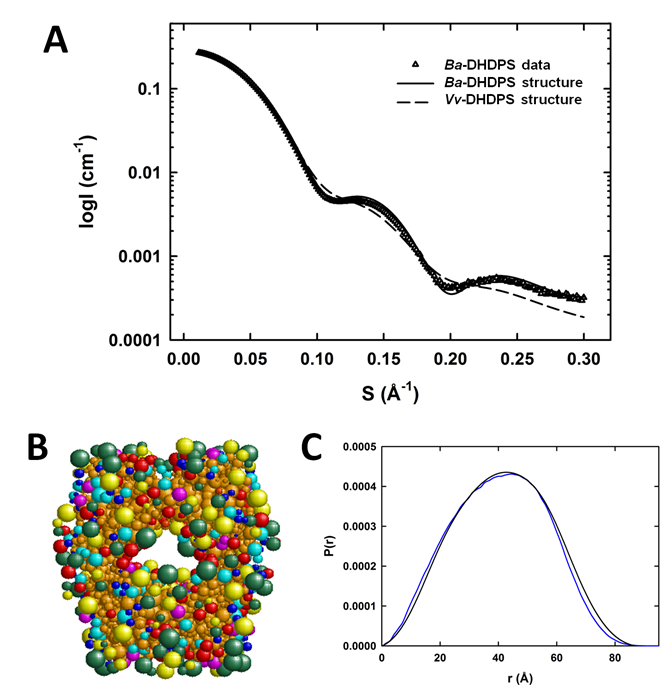

Supplement: Figure S6 — SAXS analyses of Ba -DHDPS. (A) Fits of theoretical scattering profiles from Vv-DHDPS (dashed line) and Ba-DHDPS (solid line) to the SAXS data (▴). Theoretical scattering profiles were generated from crystallographic coordinates and fitted to the Ba-DHDPS SAXS data using CRYSOL [36]. (B) SOMO bead model of Ba-DHDPS. The various colored beads represent acidic (green), hydrophobic (cyan), polar (red), basic (yellow) and non-polar (magenta) side-chains. Blue beads represent the protein main-chain and brown indicates buried beads. (C) P(r) plots of Ba-DHDPS from experimental data (black) and SOMO bead model shown in panel B (blue) (37) using ULTRASCAN [42], [43]. (TIF) [file pone.0038318.s006.tif]

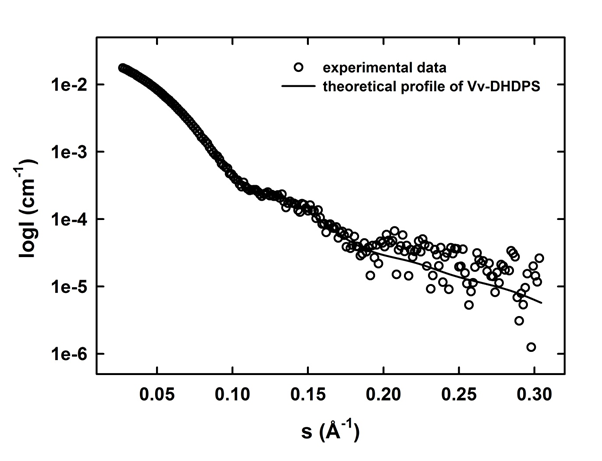

Supplement: Figure S7 — Comparison of experimental and theoretical SAXS data of Vv -DHDPS. Theoretical scattering profile from Vv-DHDPS (solid line) with 30 N-terminal residues modeled using CORAL (36) to the SAXS data (•). This fit (reduced chi-squared (χ2 v) = 1.0). This represents a statistically better fit to the data than the fit to the CRYSOL profile [(χ2 v = 1.5) (PF(F;v 1,v 2) <5.1 × 10^−11^ (F >2.2)]. (TIF) [file pone.0038318.s007.tif]

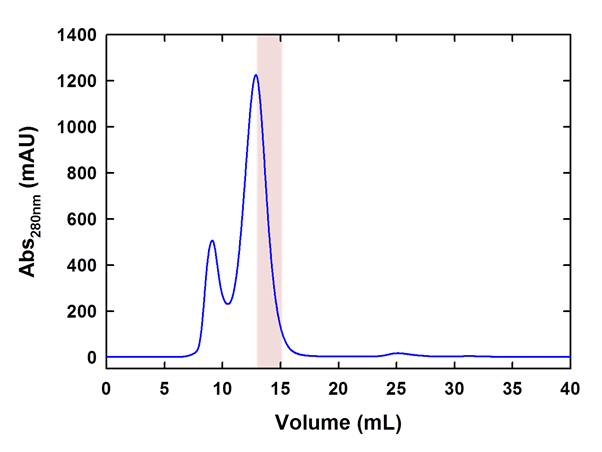

Supplement: Figure S8 — SAXS gel filtration chromatogram. Fraction employed in SAXS analysis is highlighted in pink. (TIF) [file pone.0038318.s008.tif]
