# Supplementary material for: Crystal, Solution and In silico Structural Studies of Dihydrodipicolinate Synthase from the Common Grapevine
Source: PLoS One. 2012 Jun 25;7(6):e38318. doi: 10.1371/journal.pone.0038318 (PMC3382604; doi:10.1371/journal.pone.0038318)
Supplement: Table S1 — Hydrodynamic properties of Vv -DHDPS and Ba -DHDPS. (DOCX) [file pone.0038318.s009.docx]

**Supplementary Table 1**

Hydrodynamic properties of *Vv*-DHDPS and *Ba*-DHDPS

|  | M_r_^a^ | MW^b^ | *M**^c^ | *s*_20,w_^d^ | *D*_20,w_^e^ | *f*/*f*_0_^f^ | R_0_ (Å)^g^ |
| --- | --- | --- | --- | --- | --- | --- | --- |
| 2DSA |  |  |  |  |  |  |  |
| *Vv*-DHDPS | 37876 | 151506 | 152950 | 7.38 | 0.458 | 1.35 | 35 |
| *Ba*-DHDPS | 31233 | 124932 | 116270 | 6.13 | 0.63 | 1.21 | 33 |
| SOMO |  |  |  |  |  |  |  |
| *Vv*-DHDPS | - | - | - | 7.41 | 0.521 | 1.21 | 31.9 |
| *Ba*-DHDPS | - | - | - | 7.08 | 0.524 | 1.19 | 31.3 |

^a^ Relative molecular weight calculated from amino acid sequence.

^b^ Theoretical molecular weight of DHDPS tetramer.

^c^ Apparent molecular weight derived from 2DSA analysis.

^d^ Standardized sedimentation coefficient, determined by 2DSA analysis.

^e^ Diffusion coefficient, determined from 2DSA analysis

^f^ Frictional ratio, determined from 2DSA analysis.

^g^ Equivalent radius calculated using SEDNTERP [40].
